# Supplementary material for: Breaking the cell wall for efficient DNA delivery to diatoms
Source: Nat Commun. 2026 Jan 22;17:1848. doi: 10.1038/s41467-026-68562-6 (PMC12921017; doi:10.1038/s41467-026-68562-6)
Supplement: Supplementary file 1 — Supplementary Information [file 41467_2026_68562_MOESM1_ESM.pdf]

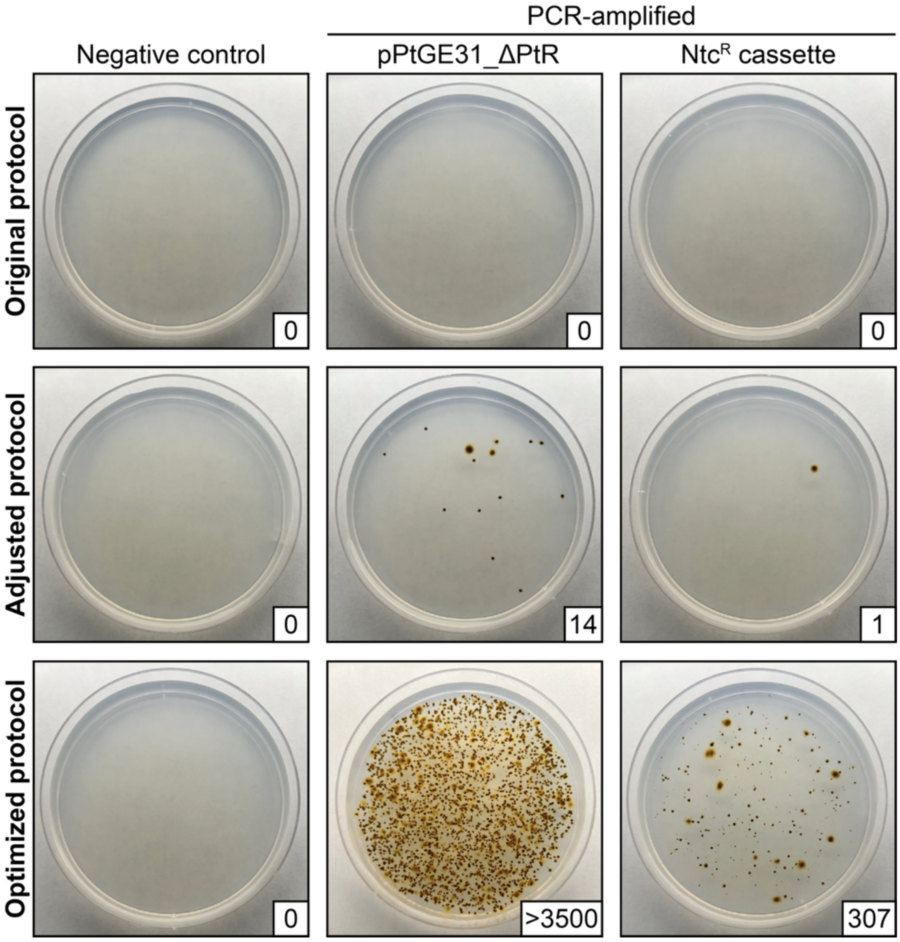

Supplementary Figure 1. A comparison between the electroporation protocols described in the main text. We began our explorations with a protocol described by Kassaw et al[1] (adapted from the methods of Zhang and Hu[2]; referred to as the original protocol). This protocol did not work in our experimental set-up until the capacitance parameter of the electroporator was adjusted from 25 µF to 50 µF (referred to as adjusted protocol). Adding an additional spheroplasting step ahead of electroporation drastically increases the efficiency for both episomal and integrative DNA (referred to as the optimized protocol). Experiments were conducted on the same day using the same number of cells (2 x 10^8 per reaction) and concentrations of DNA. The Ntc^R cassette consists of the nourseothricin resistance gene flanked by the algal FcpD/FcpA promoter/terminator pair. A quarter of the reaction was plated on ¼-salt L1 plates supplemented with 100 µg/ml nourseothricin (NTC). Equimolar amounts of episome (1 µg, 11 kb) and the cassette (~130 ng, 1.4 kb) were used for all the reactions.

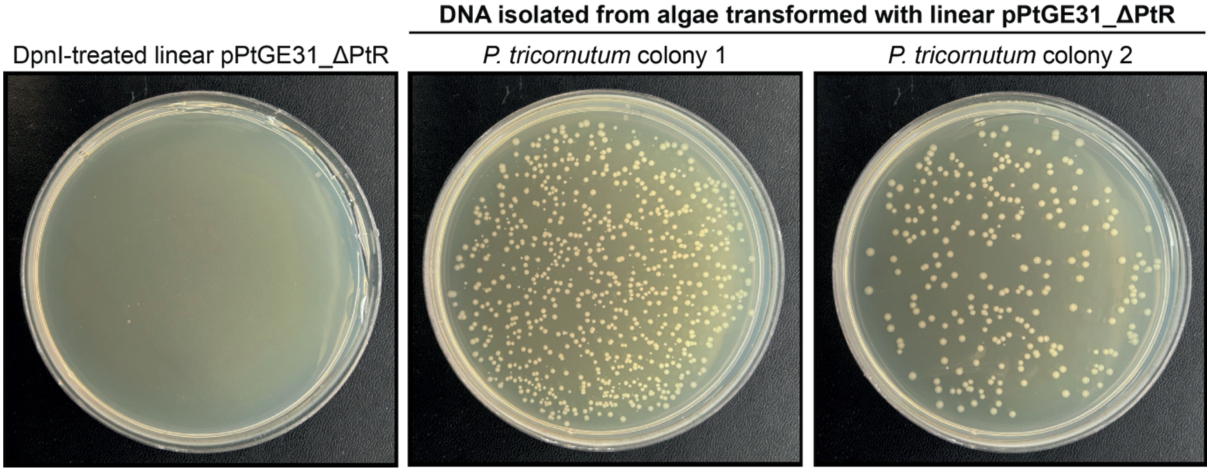

Supplementary Figure 2. Recovery of DNA from *P. tricornutum* linear pPtGE31_ΔPtR transformants using EPI300 *E. coli*. PCR-amplified and DpnI-treated pPtGE31_ΔPtR was used as a negative control to demonstrate that linear DNA cannot be propagated in EPI300.

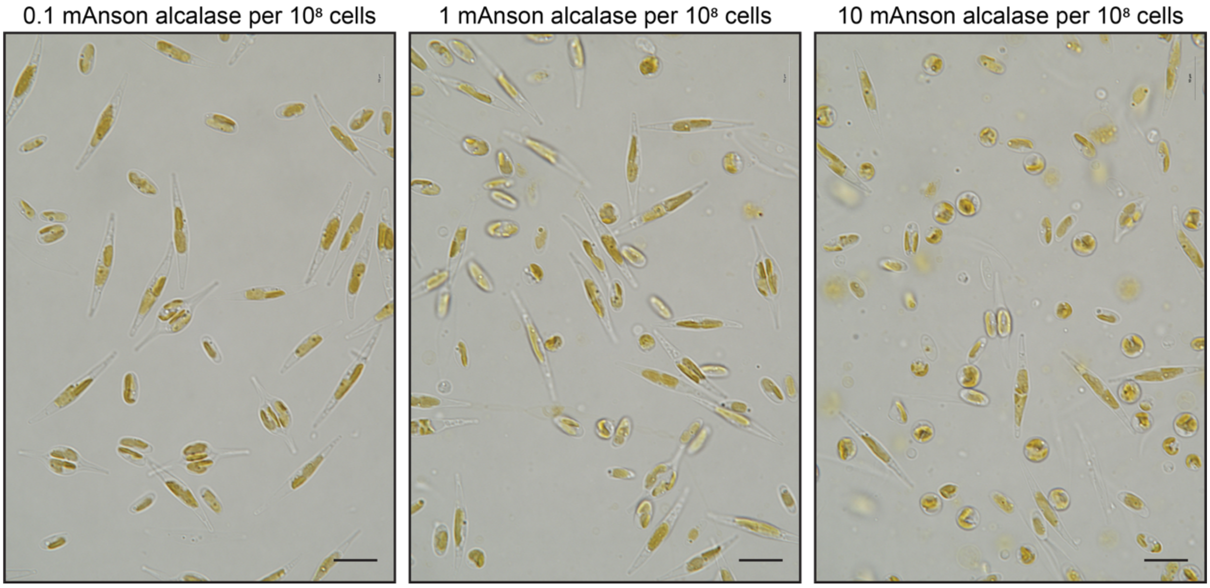

Supplementary Figure 3. Treatment of 3 × 10^8 *P. tricornutum* cells with 0.3, 3, or 30 mAnson units of alcalase. Cells were harvested from an agar plate, pelleted, and resuspended in 1 ml of 375 mM D-sorbitol prior to addition of alcalase. The treatment was carried out for 20 minutes at room temperature with gentle rocking, after which the cells were centrifuged and washed four additional times with 375 mM D-sorbitol. Microscopy was performed at 600x magnification; scale bars represent 10 µm.

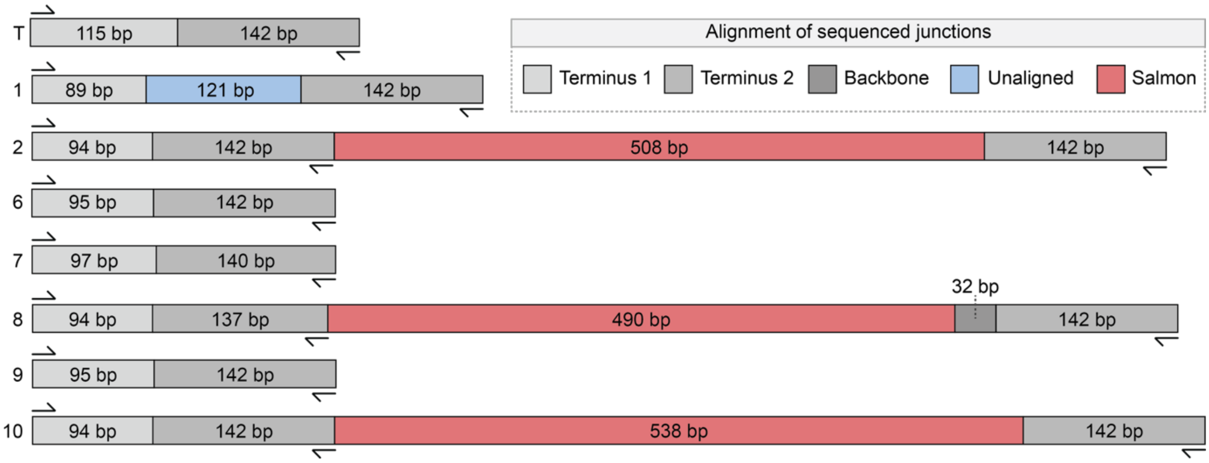

Supplementary Figure 4. Sanger sequencing of the junction region for *P. tricornutum* cells transformed with PCR-amplified pPtGE31_ ΔPtR. Sequences were aligned to the template (T), which contains the region where the episome was split during PCR-amplification. The sequencing primers were positioned ≥100 bp away from the termini to capture if any insertions or deletions were occurring in this region. Colonies 1, 2, 8, and 10 demonstrate insertions of 121 to 538 bp in size. The insertion in colony 1 did not demonstrate complementarity to any regions of pPtGE31_ ΔPtR, nor did it have any BLASTn results. The 490 to 538 bp insertions in colonies 2, 8, and 10 were queried with BLASTn and demonstrated sequence similarity (≥88%) to various regions of the salmon and/or trout genomes. Deletions of 2 to 47 bp in length occurred in colonies 6, 7, and 9.

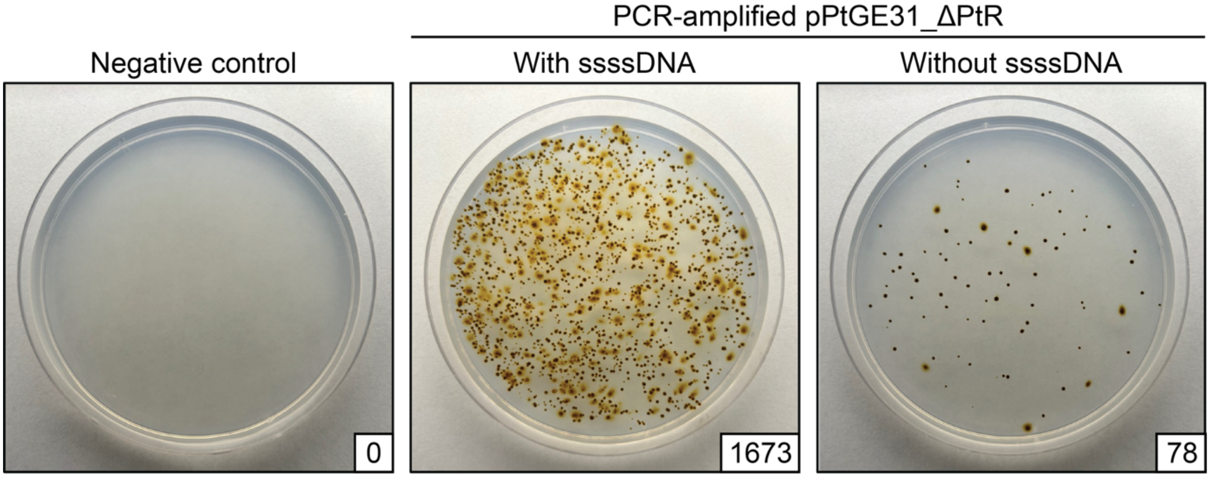

Supplementary Figure 5. Electroporation of PCR-amplified pPtGE31_ ΔPtR with and without the addition of 40 µg of single stranded salmon sperm (ssss) DNA per reaction. The number of colony forming units (CFUs) is depicted in the bottom right corner for each transformation plate. Following electroporation, one-tenth of the total reaction was plated on ¼-salt L1 plates supplemented with 100 µg/ml nourseothricin. When this experiment was repeated with three biological replicates, the difference in efficiency was approximately 15-fold, on average.

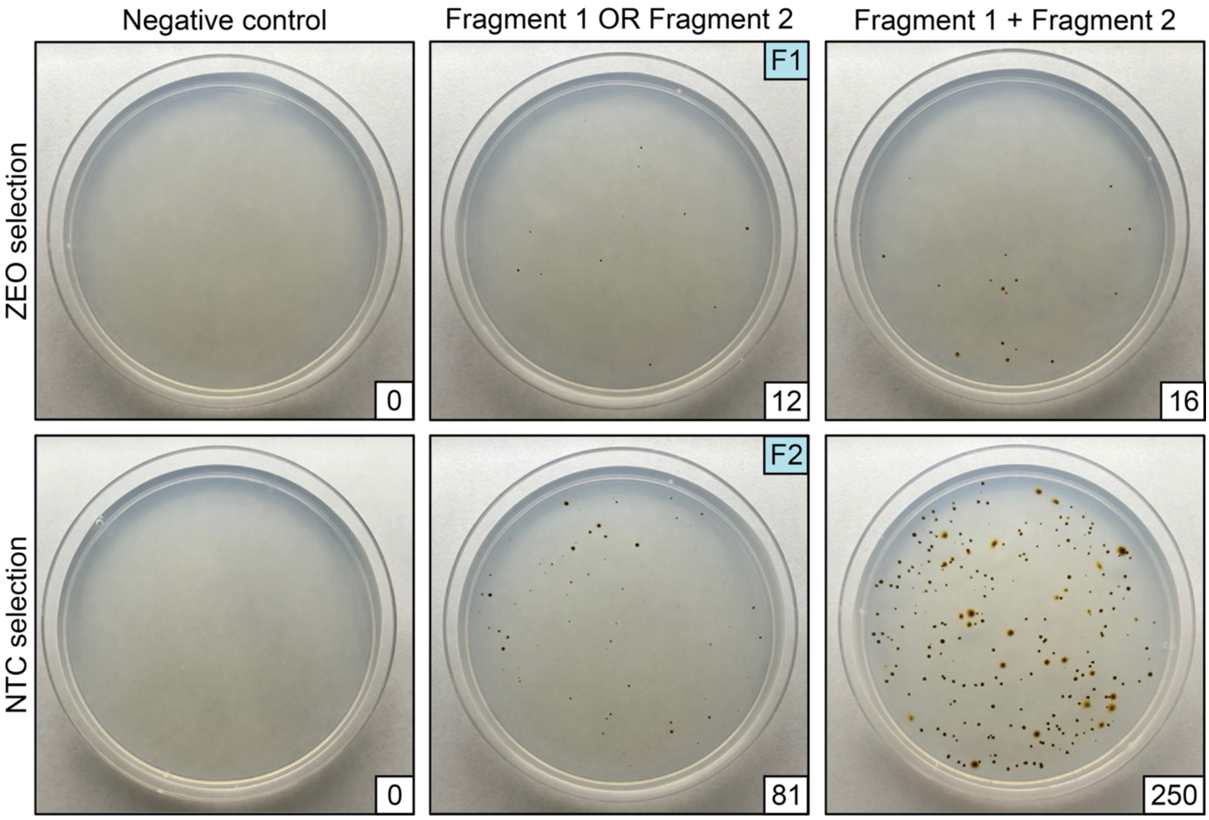

Supplementary Figure 6. Electroporation of two fragments into *P. tricornutum*. Fragment 1 contains a zeocin resistance marker, whereas fragment 2 contains a nourseothricin resistance marker. Electroporation was conducted using the individual fragments and both fragments at once. The number of colony forming units (CFUs) is depicted in the bottom right corner for each transformation plate. For each electroporation, half of the total reaction was plated on ¼-salt L1 plates supplemented with 100 µg/ml nourseothricin (NTC) or zeocin (ZEO).

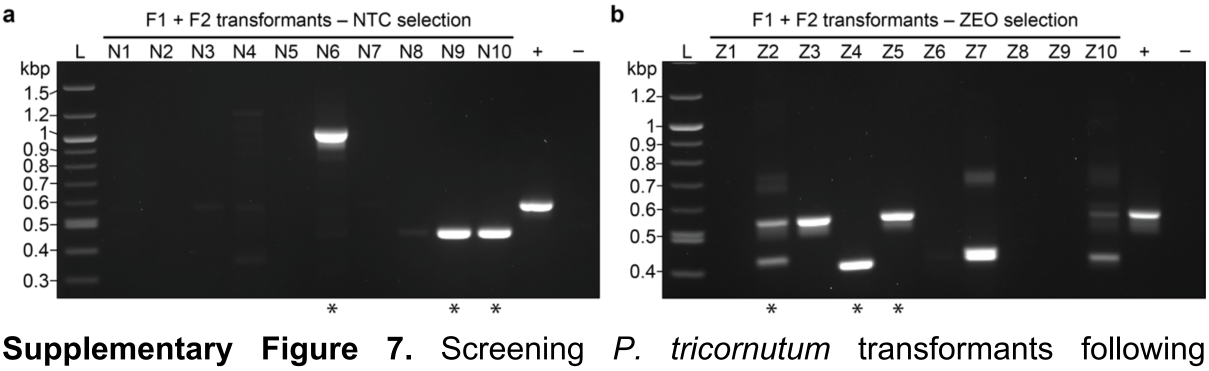

Supplementary Figure 7. Screening *P. tricornutum* transformants following electroporation with two non-overlapping fragments simultaneously. (**a**) A PCR screen for junction B across ten algal transformants that were originally passaged from the transformation plate supplemented with 100 µg/ml nourseothricin (NTC) or (**b**) 100 µg/ml zeocin (ZEO). Dilute pPtGE31_ ShBle and genomic *P. tricornutum* DNA were used for the positive and negative controls, respectively. Single asterisks (*) indicate the algal colonies that had their DNA isolated and transformed into *E. coli*.

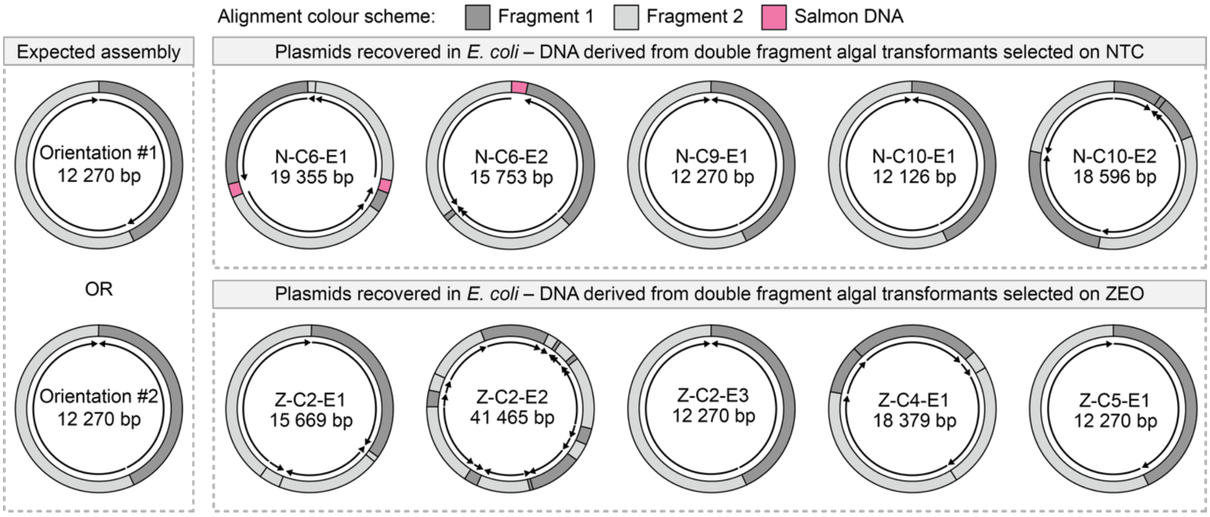

Supplementary Figure 8. Sequencing of *P. tricornutum* NHEJ-assembled episomes recovered from *E. coli* transformants. Fragment orientations are depicted by arrows and are relative to the DNA directionality in the original plasmid, pPtGE31_ShBle.

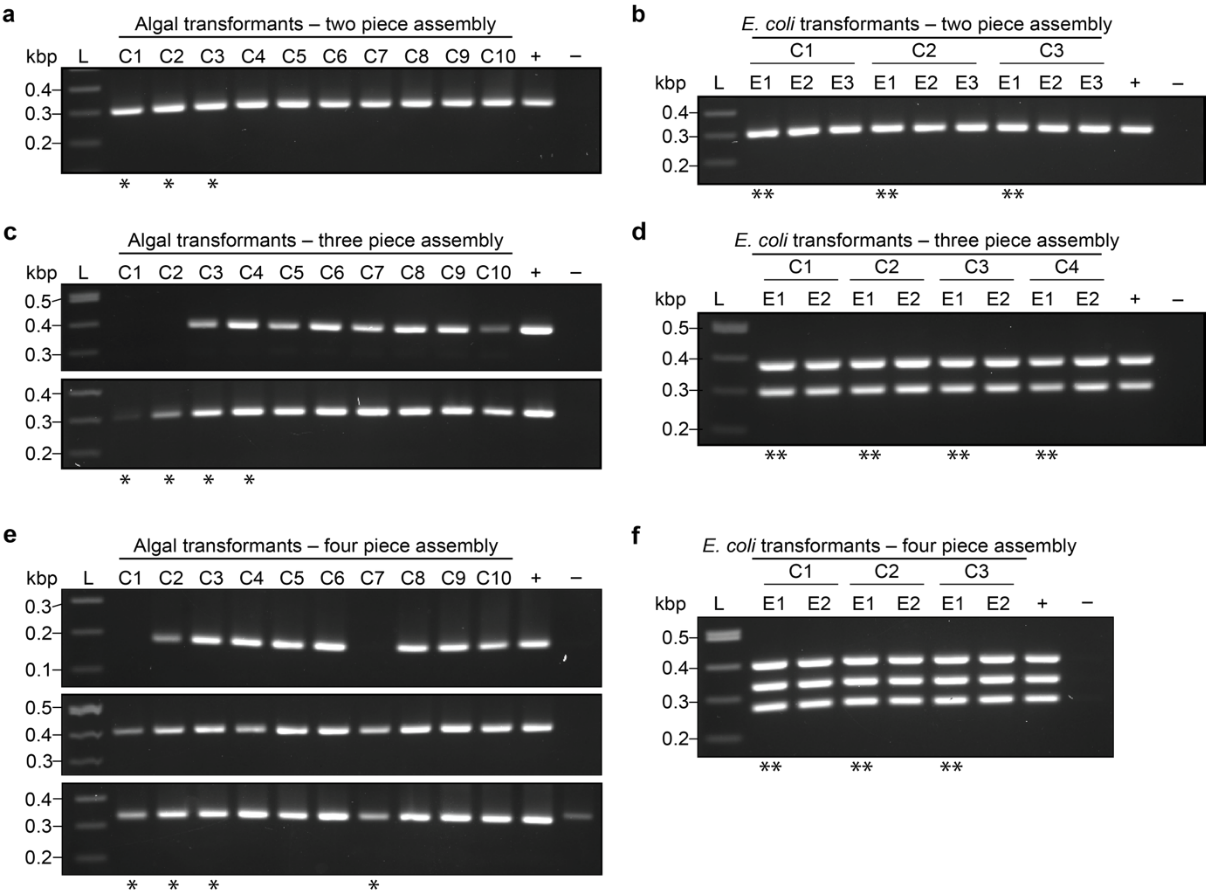

Supplementary Figure 9. PCR screening following assembly of overlapping fragments in *P. tricornutum* and recovery in *E. coli*. (**a**) Screening 10 algal transformants post electroporation with two overlapping fragments and (**b**) nine *E. coli* transformants with episomes recovered from C1, C2, and C3. (**c**) Screening 10 algal

transformants post electroporation with three overlapping fragments and (**d**) eight *E. coli* transformants with episomes recovered from C1, C2, C3 and C4. (**e**) Screening 10 algal transformants post electroporation with four overlapping fragments and (**d**) six *E. coli* transformants with episomes recovered from C1, C2, and C3. For algal screens, the positive control consists of dilute pPtGE31_ShBle, and the negative control consists of wild-type *P. tricornutum* DNA. The same positive control was used for E. coli screens, however, the negative control consisted of double-distilled water. Single asterisks (*) indicate the algal colonies that had their DNA isolated and transformed into *E. coli*. Double asterisks (**) indicate the *E. coli* colonies that were sent for whole plasmid sequencing.

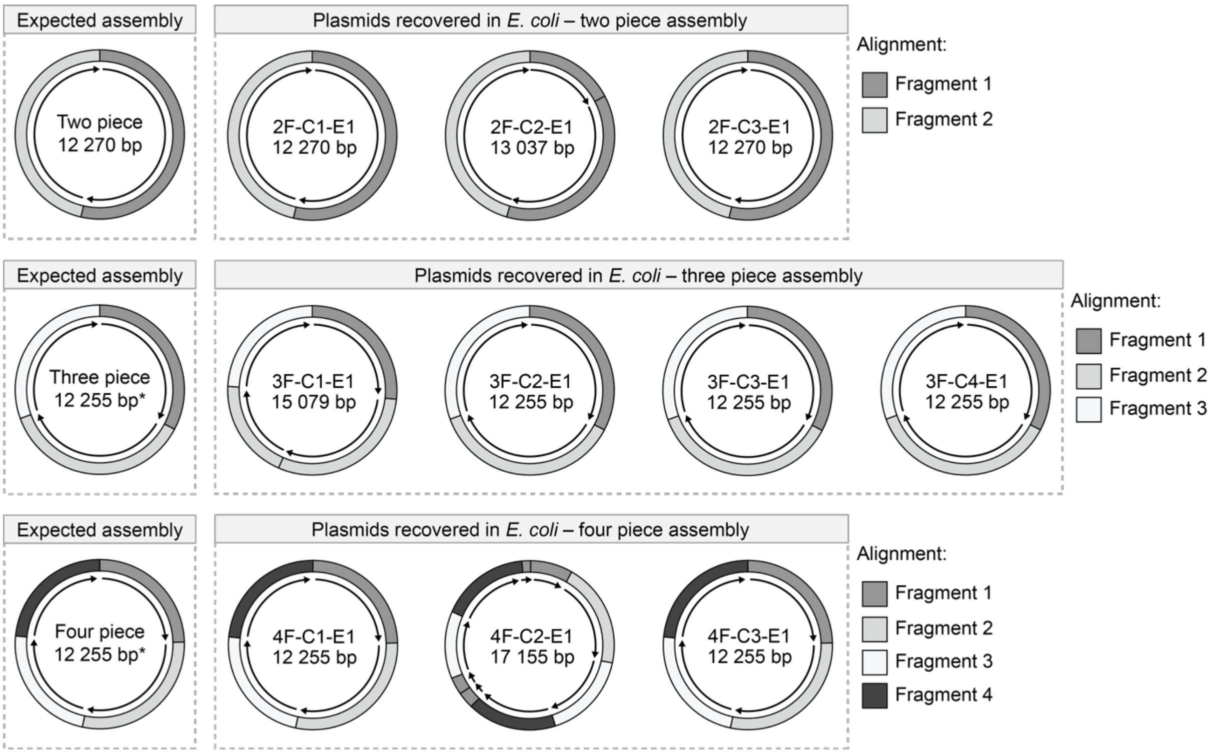

Supplementary Figure 10. Sequencing of *P. tricornutum* HR-assembled episomes recovered from *E. coli* transformants. Fragment orientations are depicted by arrows and are relative to the DNA directionality in the original plasmid, pPtGE31_ShBle. * A 15-bp deletion was consistently observed across episomes recovered from the three- and four- piece assemblies. This was determined to be due to the accidental use of pPtGE31 as template DNA during amplification of these fragments. The expected episome is 15 bp smaller than that of the two-piece assembly because pPtGE31_ShBle contains a 15 bp insertion in a non-essential region of the episomal backbone.

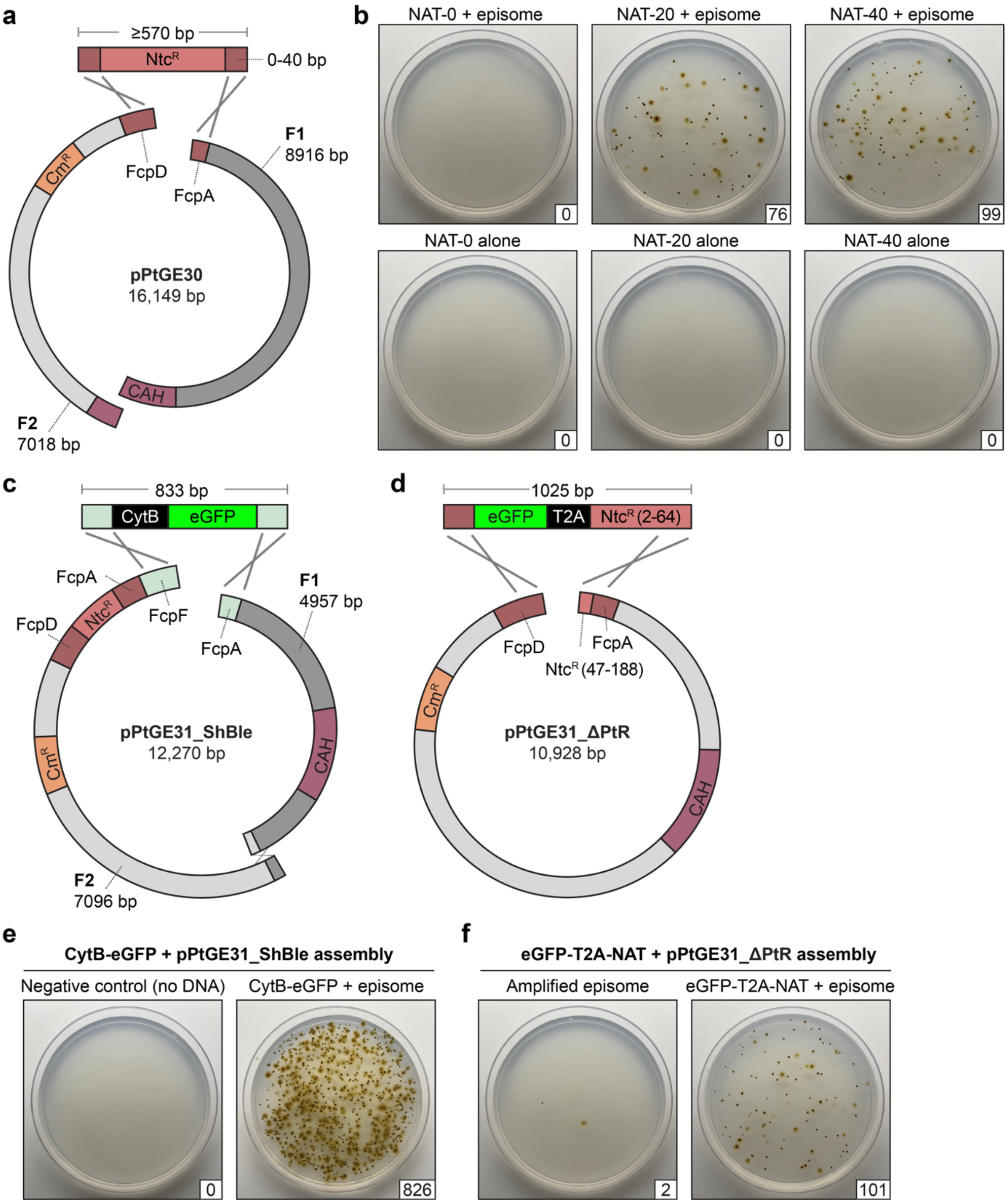

Supplementary Figure 11. Characterization of DIVA limits and applications. (a) To assess the minimal overlap required for HDR, the nourseothricin resistance marker (NtcR) was PCR-amplified with 0, 20, or 40 bp of homology to the FcpD/FcpA promoter-terminator pair and co-transformed with PCR-amplified pPtGE30 containing the *Sh Ble* (ZeoR) cassette. (b) Electroporation plates for the NAT assemblies, with or without the corresponding episomal fragments; NAT amplicons alone were tested as controls to confirm they could not confer resistance. (c) Insertion of the synthesized CytB-eGFP construct into the *Sh Ble* locus of pPtGE31_ShBle using 50 bp overlaps to the FcpF/FcpA elements. (d) Insertion of the synthesized eGFP-T2A-$NAT_{2\text{-}64}$ construct into PCR-amplified pPtGE31_ΔPtR containing an incomplete $NtcR_{47\text{-}188}$

cassette; both termini carried 50 bp of overlap to the respective insertion sites. (e) Electroporation plates for CytB-eGFP assemblies; the negative control contains spheroplasted cells electroporated without DNA. (f) Electroporation plates for the eGFP-T2A-NAT assemblies; the episome fragment alone was included as a control for template carry-over. For all electroporations, half of each reaction was plated on ¼-salt L1 agar containing 100 µg/ml nourseothricin.

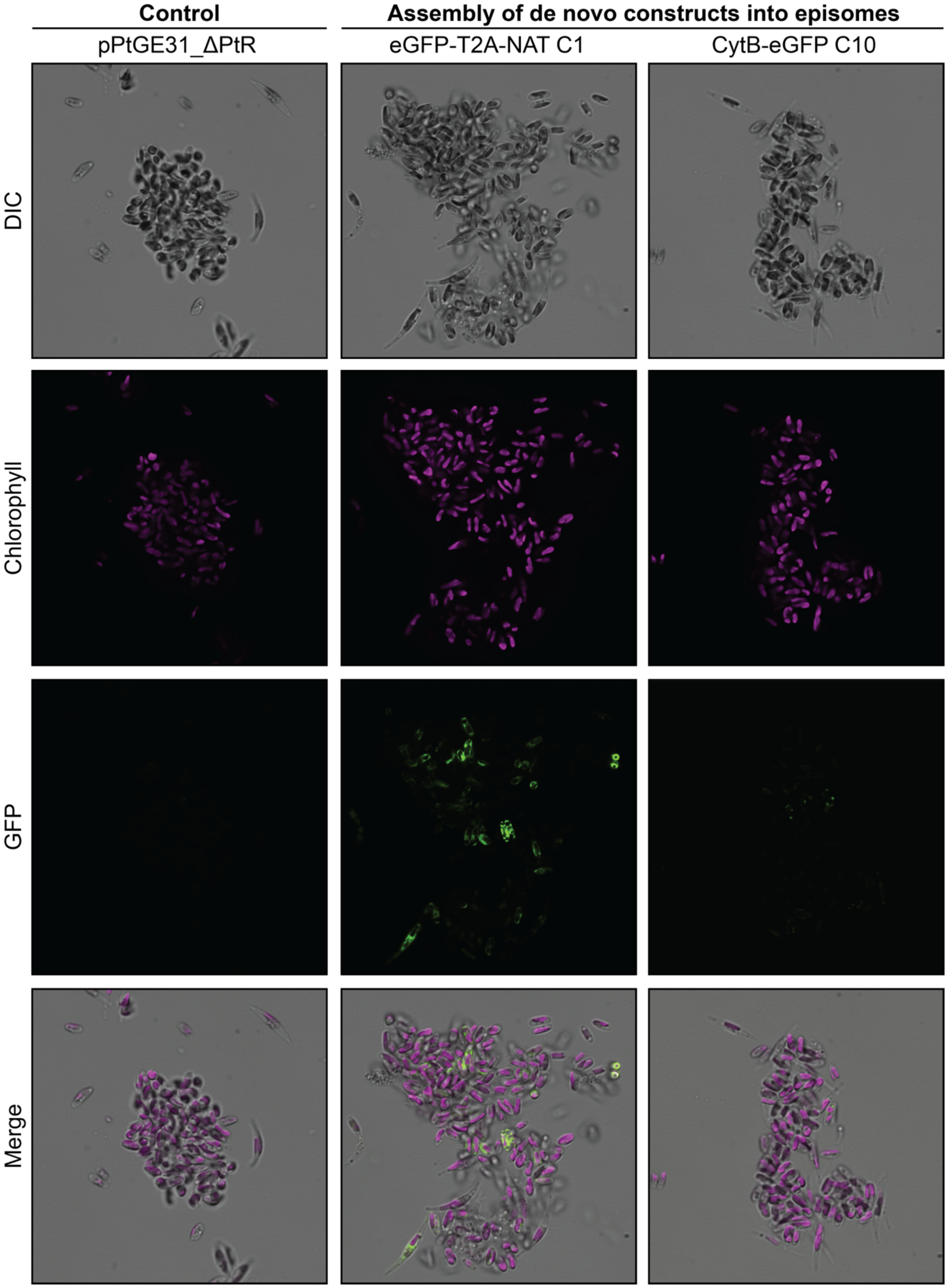

Supplementary Figure 12. Confocal fluorescence microscopy of *P. tricornutum* electroporation transformants. A strain harboring pPtGE31_ΔPtR, which lacks an eGFP marker, was used as a control. The other two colonies were isolated following transformation with the eGFP-T2A-NAT and CytB-eGFP constructs, which are expected to express eGFP in the cytosol and mitochondria, respectively, if assembled

correctly into their respective episomes. Chlorophyll channel excitation/emission wavelengths: 405 and 488/691 nm; GFP channel excitation/emission wavelengths: 488/531 nm.

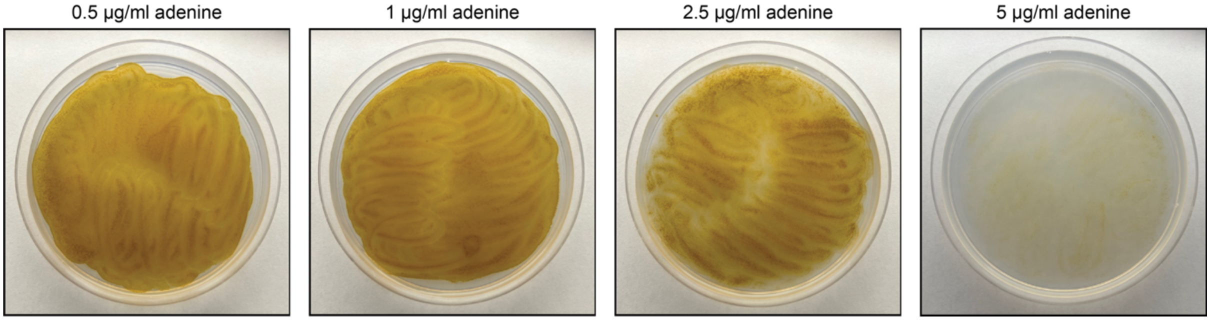

Supplementary Figure 13. The growth of wild-type *P. tricornutum* on ½-salt L1 plates supplemented with differing amounts of adenine. Approximately 2.5 x 10^7 cells (250 µl at a concentration of 1 x 10^8 cells/ml) were spread onto each plate, which were then placed in a growth chamber for 1 week before being pictured.

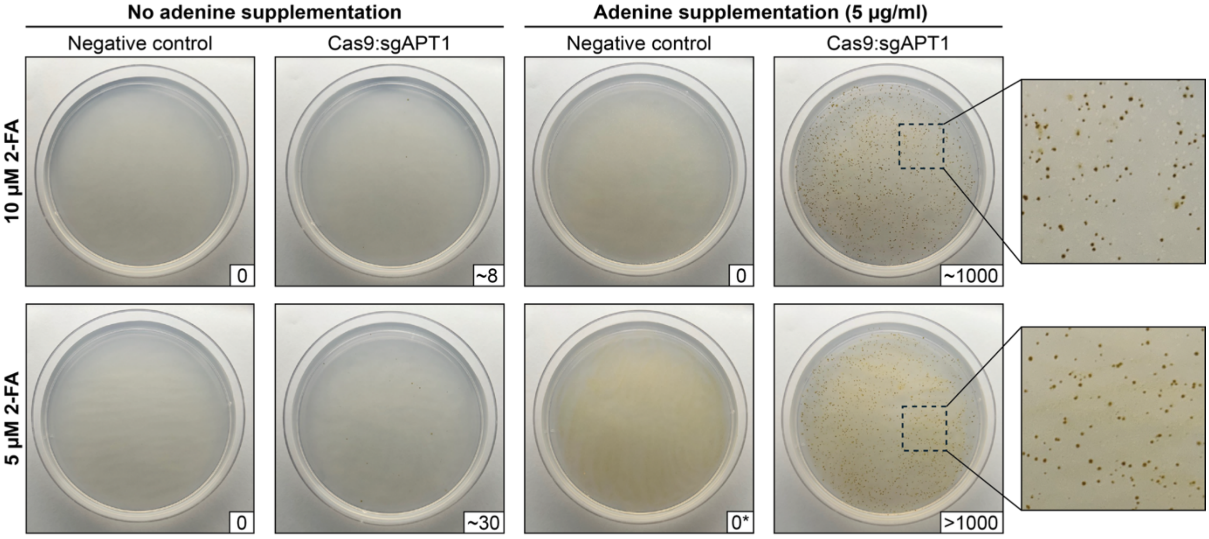

Supplementary Figure 14. Electroporating an RNP complex (Cas9:sgAPT1) into spheroplasted *P. tricornutum* cells. Successful knockout of the PtAPT loci generates colonies that are resistant to 2-fluoroadenine (2-FA). One-fifth of the total reaction was plated across ½ salt L1 plates supplemented with 10 or 5 µM 2-FA and with or without an additional 5 µg/ml adenine. Plates were removed from the growth chamber and pictured 12 days post-electroporation to prevent breakthrough from ultraviolet irradiation degradation of the 2-FA. *No individual colonies were visible on this plate, however, a thin layer of algal growth is apparent.

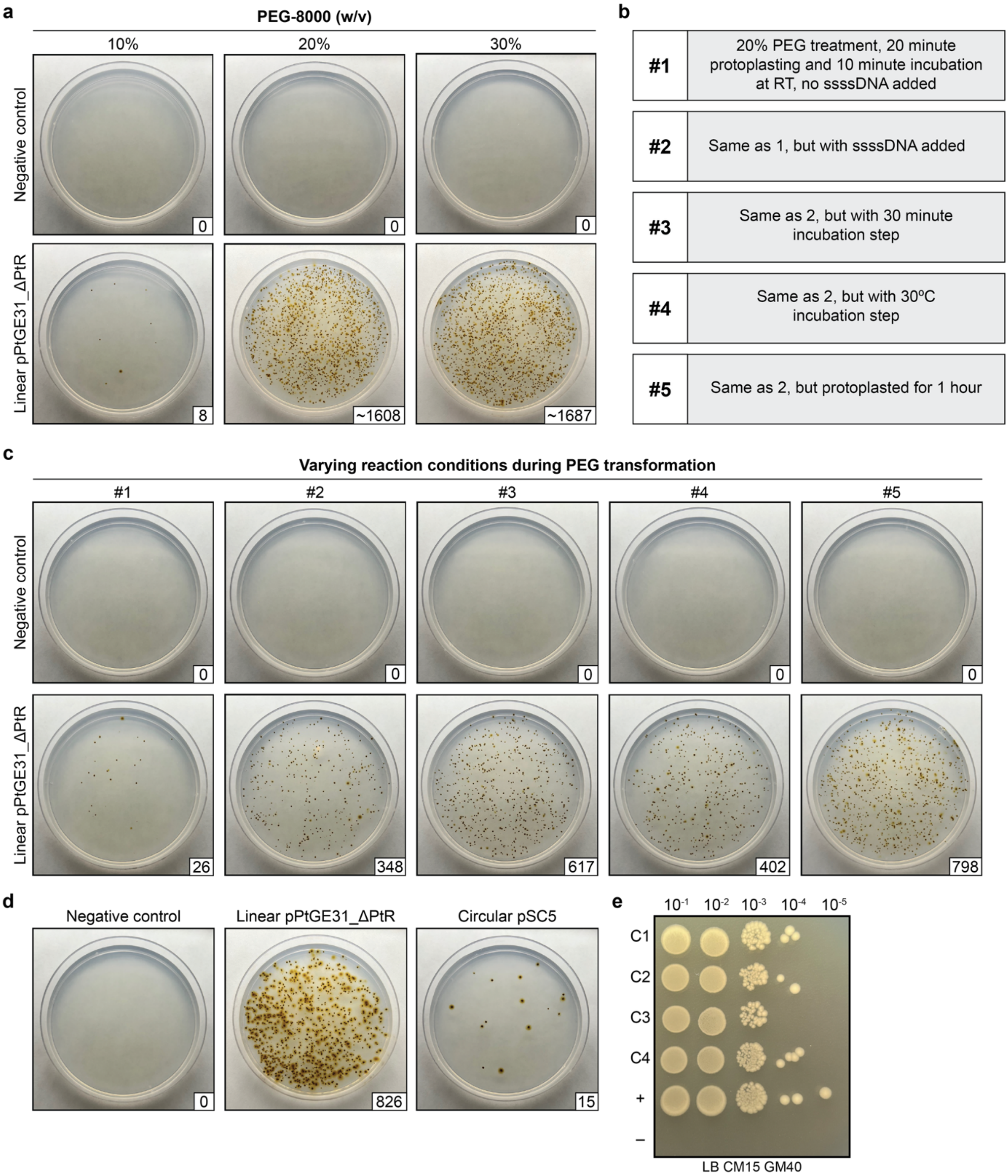

Supplementary Figure 15. Establishing an efficient chemical transformation method for *P. tricornutum*. (**a**) Testing the transformation efficiency with different concentrations (w/v) of polyethylene glycol (PEG) 8000. (**b**) Five different experiments were carried out simultaneously, where one or more variables of the transformation method were changed. (**c**) The resulting transformation plates from the experimental conditions listed in b. (**d**) Transforming a 55.6 kb episome, pSC5, through the PEG method. (**e**) Testing the conjugation efficiency of *E. coli* strains harbouring episomes that had been recovered from *P. tricornutum* PEG transformants. Strains harbouring pSC5 or pSAP were used as the positive and negative controls, respectively. Dilutions of 10^{-1} to 10^{-5} were spot plated on LB plates supplemented with 15 µg/ml chloramphenicol and 40 µg/ml gentamicin. For experiments in panel a and d, a fourth

of the PEG reaction was plated onto ¼-salt L1 plates supplemented with nourseothricin (100 µg/ml). In panel c, half of the PEG reaction was plated onto the same type of selection plates.

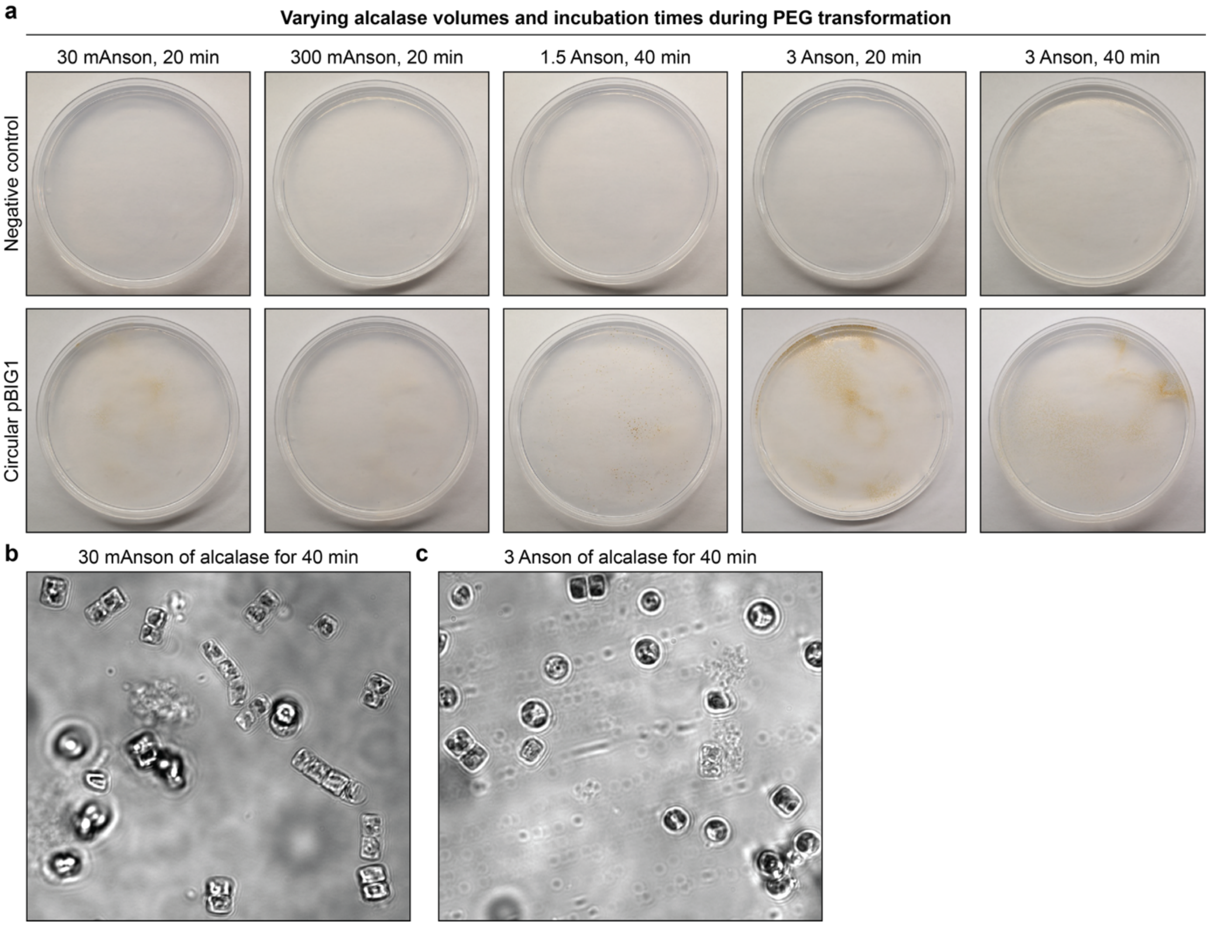

Supplementary Figure 16. PEG transformation of *T. pseudonana*. (**a**) Harvested cells were subjected to differing amounts of alcalase and lengths of incubation during the protoplasting step of the PEG transformation method. Following recovery, half of the total reaction was plated on full-salt F/2 plates supplemented with 100 µg/ml nourseothricin. (**b**) Alcalase treatment of *T. pseudonana* with 10 µl (30 mAnson) or (**c**) 1000 µl (3 Anson) of alcalase for 40 minutes.

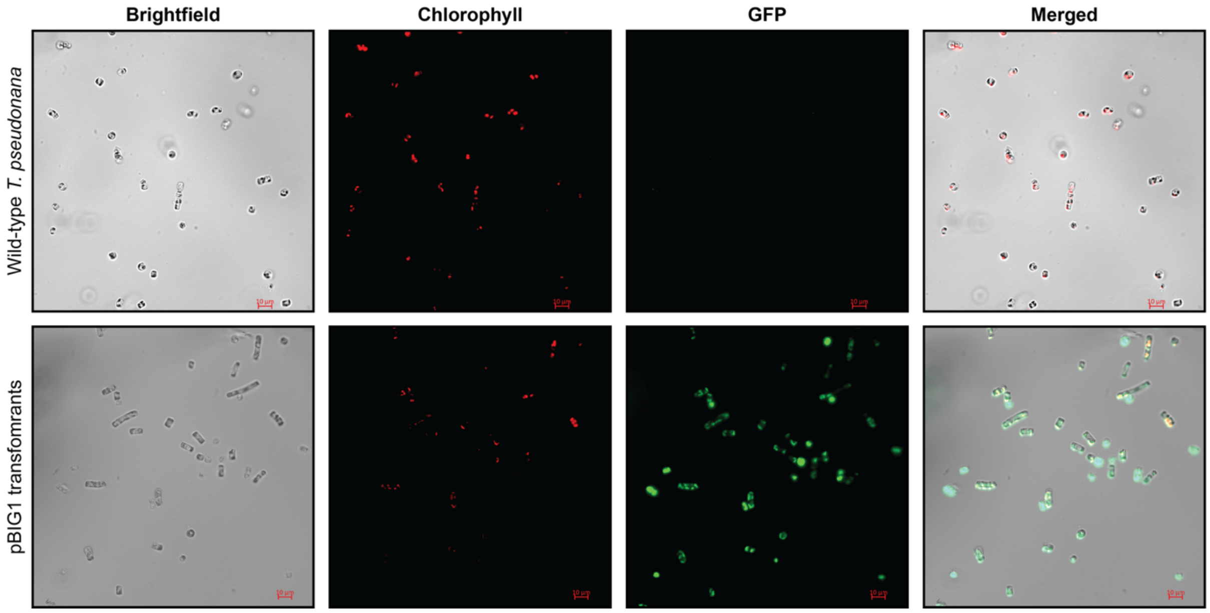

Supplementary Figure 17. Fluorescence microscopy of wild-type (i.e., untransformed) and PEG-transformed *T. pseudonana* cells. Cells were transformed with pBIG1, which contains eGFP.

Supplementary Table 1. Transformation efficiencies for *P. tricornutum* cells cultured in liquid media or on agar plates, with or without alcalase treatment. Data were analyzed from five biological replicates, with there being an average of 1.8 x 10^8 cells per transformation reaction. The values for mean CFUs were rounded to the nearest whole integer; all other values were rounded to three significant digits.

Media and treatment	Mean CFUs per reaction	Standard error of the mean	Efficiency
Liquid, untreated	76	19.9	4.22 x 10^7
Liquid, treated	280	137.8	1.56 x 10^6
Plated, untreated	108	43.0	6.02 x 10^7
Plated, treated	21,495	5.44 x 10^3	1.19 x 10^4

Supplementary Table 2. Transformation efficiencies for *P. tricornutum* cells transformed with differing amounts of PCR-amplified pPtGE31_ ΔPtR. Data were analyzed from four biological replicates, with there being an average of 1.24 x 10^8 cells per transformation reaction. The values for mean CFUs per reaction and per ng of DNA were rounded to the nearest whole integer; the standard error of the mean values were rounded to three significant digits.

Amount of DNA	Mean CFUs per reaction	Standard error of the mean	CFUs per ng DNA

1 ng	7	4.04	7
10 ng	372	136	37
100 ng	1545	536	15
500 ng	13,109	5.38 x 10^3	26
1000 ng	16,629	6.68 x 10^3	17

Supplementary Table 3. Passaging *P. tricornutum* pPtGE31_ ΔPtR transformants from Fig. 2D-E with or without selection. Cultures were passaged in liquid media, with or without nourseothricin selection, seven times and then serially diluted onto non-selective media to obtain single colonies. For each transformant, 100 single colonies were then patched onto non-selective media. After 1 week of growth, colonies were repatched onto selective media.

**Algal transformant**	**Proportion of nourseothricin resistant colonies**	
	Passaged without selection	Passaged with selection
Circular plasmid, transformant 1	7/100	36/100
Circular plasmid, transformant 2	4/100	48/100
Linear plasmid, transformant 1	6/100	40/100
Linear plasmid, transformant 8	2/100	47/100

Supplementary Table 4. Passaging of *P. tricornutum* colonies transformed with two fragments, both individually and simultaneously. Transformants were initially replated on ¼-salt L1 plates supplemented with the same antibiotic selection as the original transformation plate (N = 100 µg/ml nourseothricin, Z = 100 µg/ml zeocin). On the second passage, colonies were repatched onto ¼-salt L1 plates containing the alternative antibiotic selection to screen for the presence of both resistance marker cassettes. Fragment 1 carries the zeocin-resistance marker, whereas fragment 2 carries the nourseothricin-resistance marker. Single fragment transformants were only plated on one type of selection plate.

**Fragment(s)**	**Initial colonies**		**First passage**		**Second passage**	
	**N**	**Z**	**N → N**	**Z → Z**	**N → N → Z**	**Z → Z → N**
Fragment 1	N/A	12	N/A	10/12	N/A	0/10
Fragment 2	81	N/A	12/20	N/A	0/12	N/A
Both fragments	250	16	100/100	16/16	79/100	15/16

Supplementary Table 5. Passaging of *P. tricornutum* colonies co-transformed with pPtGE31_ΔPtR and Cas9:sgAPT1. One-fifth of the transformation volume was plated across two selection types: (1) ½-salt L1 plates supplemented with 5 μg/ml adenine and 10 μM 2-FA, and (2) ½-salt L1 plates supplemented with 1 μg/ml adenine and 100 μg/ml nourseothricin. Fifty colonies were first passaged from each selection plate onto the same selection type. On the second passage, colonies were repatched onto the other selection type.

Selection type	Initial colonies	First passage	Second passage
NAT	80	50/50	7/50
2-FA	50	48/50	6/50

SUPPLEMENTARY REFERENCES

1. Kassaw, T. K., Paton, A. J. & Peers, G. Episome-Based Gene Expression Modulation Platform in the Model Diatom *Phaeodactylum tricornutum*. *ACS Synth. Biol.* **11**, 191–204 (2022).
2. Zhang, C. & Hu, H. High-efficiency nuclear transformation of the diatom *Phaeodactylum tricornutum* by electroporation. *Marine Genomics* **16**, 63–66 (2014).
