# Supplementary material for: Breaking the cell wall for efficient DNA delivery to diatoms
Source: Nat Commun. 2026 Jan 22;17:1848. doi: 10.1038/s41467-026-68562-6 (PMC12921017; doi:10.1038/s41467-026-68562-6)
Supplement: Supplementary file 2 — Description of Additional Supplementary Information [file 41467_2026_68562_MOESM2_ESM.docx]

**Description of Additional Supplementary Files**

File Name: Supplementary Data 1

Description: Initial optimization of electroporation parameters for P. *tricornutum*. Each experiment used 1.0 – 2.0 x 10^6^ cells that had been harvested from liquid cultures. For experiments 60 to 74, transformants were plated on nourseothricin (NTC) and zeocin (ZEO) selection; NTC selection was used for all other experiments. The marker cassettes were generated through PCR amplification, whereas plasmids were purified from *E. coli*. Linear plasmid DNA was obtained through restriction digest with a single-cutter enzyme (exp. 1 – 6 and 18 – 22) or through PCR amplification (exp. 81 – 89). Intact circular plasmid DNA was used in experiments 77 – 79. EE: early exponential growth phase, LS: late stationary growth phase

File Name: Supplementary Data 2

Description: Oligonucleotides used in this study.

File Name: Supplementary Data 3

Description: FASTA file containing the Sanger sequences produced from this study.

File Name: Supplementary Data 4

Description: FASTA file containing the Oxford Nanopore whole plasmid/episome sequences produced from this study.

File Name: Supplementary Data 5

Description: FASTA file containing the sequences of the eGFP-T2A-NAT and CytB-eGFP gBlocks that were synthesized for this study.
