# Supplementary material for: Breaking the cell wall for efficient DNA delivery to diatoms
Source: Nat Commun. 2026 Jan 22;17:1848. doi: 10.1038/s41467-026-68562-6 (PMC12921017; doi:10.1038/s41467-026-68562-6)
Supplement: Supplementary file 8 — Reporting Summary [file 41467_2026_68562_MOESM8_ESM.pdf]

nature portfolio

Corresponding author(s): Bogumil Karas

Last updated by author(s): Dec 31, 2025

Reporting Summary

Nature Portfolio wishes to improve the reproducibility of the work that we publish. This form provides structure for consistency and transparency in reporting. For further information on Nature Portfolio policies, see our Editorial Policies and the Editorial Policy Checklist.

Statistics

For all statistical analyses, confirm that the following items are present in the figure legend, table legend, main text, or Methods section.

n/a	Confirmed	
☐	☒	The exact sample size (*n*) for each experimental group/condition, given as a discrete number and unit of measurement
☐	☒	A statement on whether measurements were taken from distinct samples or whether the same sample was measured repeatedly
☐	☒	The statistical test(s) used AND whether they are one- or two-sided<br>*Only common tests should be described solely by name; describe more complex techniques in the Methods section.*
☒	☐	A description of all covariates tested
☒	☐	A description of any assumptions or corrections, such as tests of normality and adjustment for multiple comparisons
☐	☒	A full description of the statistical parameters including central tendency (e.g. means) or other basic estimates (e.g. regression coefficient) AND variation (e.g. standard deviation) or associated estimates of uncertainty (e.g. confidence intervals)
☐	☒	For null hypothesis testing, the test statistic (e.g. *F*, *t*, *r*) with confidence intervals, effect sizes, degrees of freedom and *P* value noted<br>*Give P values as exact values whenever suitable.*
☒	☐	For Bayesian analysis, information on the choice of priors and Markov chain Monte Carlo settings
☒	☐	For hierarchical and complex designs, identification of the appropriate level for tests and full reporting of outcomes
☒	☐	Estimates of effect sizes (e.g. Cohen's *d*, Pearson's *r*), indicating how they were calculated

Our web collection on statistics for biologists contains articles on many of the points above.

Software and code

Policy information about availability of computer code

Data collection	*Provide a description of all commercial, open source and custom code used to collect the data in this study, specifying the version used OR state that no software was used.*
Data analysis	Primer3, RStudio, Excel

For manuscripts utilizing custom algorithms or software that are central to the research but not yet described in published literature, software must be made available to editors and reviewers. We strongly encourage code deposition in a community repository (e.g. GitHub). See the Nature Portfolio guidelines for submitting code & software for further information.

Data

Policy information about availability of data

All manuscripts must include a data availability statement. This statement should provide the following information, where applicable:

- Accession codes, unique identifiers, or web links for publicly available datasets
- A description of any restrictions on data availability
- For clinical datasets or third party data, please ensure that the statement adheres to our policy

We added this text to manuscript: Sequencing information is available in the Supplementary Datasets 3, 4, and 5. The raw P. tricornutum colony counts used to estimate transformation efficiency for the graphs in Fig. 1E-F are available in the Source Data File. All constructs used in P. tricornutum are also deposited to Addgene, as referenced throughout the text.

Research involving human participants, their data, or biological material

Policy information about studies with human participants or human data. See also policy information about sex, gender (identity/presentation), and sexual orientation and race, ethnicity and racism.

Reporting on sex and gender	Does not apply
Reporting on race, ethnicity, or other socially relevant groupings	Does not apply
Population characteristics	Does not apply
Recruitment	Does not apply
Ethics oversight	Does not apply

Note that full information on the approval of the study protocol must also be provided in the manuscript.

Field-specific reporting

Please select the one below that is the best fit for your research. If you are not sure, read the appropriate sections before making your selection.

[x] Life sciences [ ] Behavioural & social sciences [ ] Ecological, evolutionary & environmental sciences

For a reference copy of the document with all sections, see nature.com/documents/nr-reporting-summary-flat.pdf

Life sciences study design

All studies must disclose on these points even when the disclosure is negative.

Sample size	No formal statistical power calculations were performed prior to experimentation. For experiments intended to assess trends or allow statistical comparisons (e.g., Fig. 1E and 1F), at least four independent biological replicates were performed to account for experiment-to-experiment variability in transformation efficiency. This exceeds the commonly used minimum of three biological replicates and was chosen to improve robustness of the estimates.<br><br>For all other experiments, which were qualitative or proof-of-concept in nature (e.g., episome assembly, RNP delivery), sample sizes were selected to demonstrate reproducibility rather than statistical significance, and at least two independent biological replicates were performed. These sample sizes were sufficient to support the conclusions drawn, which were based on clear, objective experimental outcomes rather than quantitative comparisons.
Data exclusions	No data was excluded
Replication	All attempts at replication have been successful.
Randomization	Samples were not randomly allocated as this study focused on method development and proof-of-concept experiments. Experimental groups were defined by the transformation condition being tested and processed in parallel to minimize any sources of variation (i.e., same batch of prepared cells were used for all transformations in a given experiment).
Blinding	Blinding was not relevant for this study because the experiments were primarily methodological and proof-of-concept in nature, with outcomes based on objective and directly observable results (e.g., emergence of colonies, presence or absence of fluorescence, and PCR screening episomes, sequencing, etc).

Reporting for specific materials, systems and methods

We require information from authors about some types of materials, experimental systems and methods used in many studies. Here, indicate whether each material, system or method listed is relevant to your study. If you are not sure if a list item applies to your research, read the appropriate section before selecting a response.

Materials & experimental systems

n/a	Involved in the study
☒	☐ Antibodies
☒	☐ Eukaryotic cell lines
☒	☐ Palaeontology and archaeology
☒	☐ Animals and other organisms
☒	☐ Clinical data
☒	☐ Dual use research of concern
☒	☐ Plants

Methods

n/a	Involved in the study
☒	☐ ChIP-seq
☒	☐ Flow cytometry
☒	☐ MRI-based neuroimaging

Plants

Seed stocks	Does not apply
Novel plant genotypes	Does not apply
Authentication	Does not apply
